# Haematological and electrophoretic characterisation of β-thalassaemia in Yunnan province of Southwestern China

**DOI:** 10.1136/bmjopen-2016-013367

**Published:** 2017-01-31

**Authors:** Jie Zhang, Jing He, Xiaoqin Mao, Xiaohong Zeng, Hong Chen, Jie Su, Baosheng Zhu

**Affiliations:** 1 Yunnan Provincial Key Laboratory For Birth Defects and Genetic Diseases, Genetic Diagnosis Center, the First People's Hospital of Yunnan Province, Kunming, China; 2 Genetics Department, Kunming University of Science and Technology; 3 Department of Clinical Laboratory, The First People's Hospital of Yunnan Province, Kunming, China

**Keywords:** thalassemia, capillary electrophoresis, hematological and biochemical parameter values, mutation, cut-off value

## Abstract

**Objectives:**

β-Thalassaemia is widely found in Southwestern China. Characterisation of β-thalassaemia can improve screening and prenatal diagnosis for at-risk populations.

**Design:**

A retrospective study.

**Methods:**

In this study, the levels of haemoglobin alpha 2 (HbA_2_) and haemoglobin alpha (HbA) were analysed by gender for a total of 15 067 subjects screened by capillary electrophoresis. The cut-off value with the highest accuracy was established to identify β-thalassaemia in 723 patients suspected to have this disease. Haematological and electrophoretic characterisation of eight common types of β-thalassaemia were analysed in 486 β-thalassaemia subjects.

**Results:**

HbA levels were significantly higher in men than in women, but there was no significant difference on HbA_2_ levels. A new cut-off value for the diagnosis of β-thalassaemia (HbA_2_≥4.0%) with the highest accuracy was proposed for the studied populations. Haemoglobin (Hb) was significantly higher in men compared with women (p<0.05), whereas no statistically significant differences were found for mean cell volume (MCV), mean cell haemoglobin (MCH), HbA and HbA_2_. The haemoglobin E (HbE) group showed comparatively higher values for haematological indices (Hb, MCV and MCH) than the other genotypes in heterozygous β-thalassaemia groups (p<0.05), and −28 (A>G) (HBB (β-globin):c.−78A>C) had signiﬁcantly higher HbA_2_ values compared with other β-thalassaemia.

**Conclusions:**

Ethnic groups have diversified β-globin gene mutations and considerable haematological variations. Our study will lay the foundation for screening programmes and clinical management of thalassaemia in Southwestern China.

**Strengths and limitations of this study**

- Ours is the first study to characterise β-thalassaemia in the Yunnan province, which may be useful to improve screening and prenatal diagnosis in Southwestern China.
- Our study determined that a higher cut-off point of haemoglobin alpha 2 levels (HbA_2_ ≥4.0%) should be used for β-thalassaemia screening, rather than the current value of 3.5%.
- Samples for participants used for capillary electrophoresis, cut-off value calculation and characterisation of β-thalassaemia were not collected at the same time.

## Introduction

β-Thalassaemia is an inherited anaemia resulting from genome variants in β-globin chains. This disease is most prevalent in Africa,^1^ Asia,^2^ Mediterranean^3^ and the Middle East.^4^ Quantification of haemoglobin alpha 2 (HbA_2_) percentage by capillary electrophoresis and routine haematology testing are the existing methods for screening thalassaemia and haemoglobinopathies.^5^ An HbA_2_ value exceeding 3.5% combined with a low mean cell volume (MCV) and mean cell haemoglobin (MCH) are the current diagnostic criteria for β-thalassaemia carriers. Determination of the haemoglobin (Hb) concentration and HbA_2_ range in a population offers a critical screening tool for thalassaemia.^6^ ^7^ However, there are very few reports on the clinical reference intervals for β-thalassaemia patients in China. Therefore, it is important to determine appropriate diagnostic cut-off points and supplement the existing references to improve the screening and control of β-thalassaemia.

More than 800 different β-globin gene mutations have been discovered worldwide. Two subtypes are defined by totally absent (β^0^) or partially reduced (β^+^) production of normal β chains, respectively. The severity of β-thalassaemia varies with the thalassaemia mutation, ranging from asymptomatic anaemia to a severe transfusion-dependent disorder. Populations in different regions have diversified β-globin gene mutations^8^ and the ability to identify and characterise these β-thalassaemia mutations can assist in genetic counselling and prenatal diagnoses.^9^ In this study, we established an HbA_2_ data set for screening β-thalassaemia in people of Southwestern China, and correlated these data to haematological and biochemical values to better understand thalassaemia and to improve its control and prevention.

## Materials and methods

### Study design

**Figure 1:**
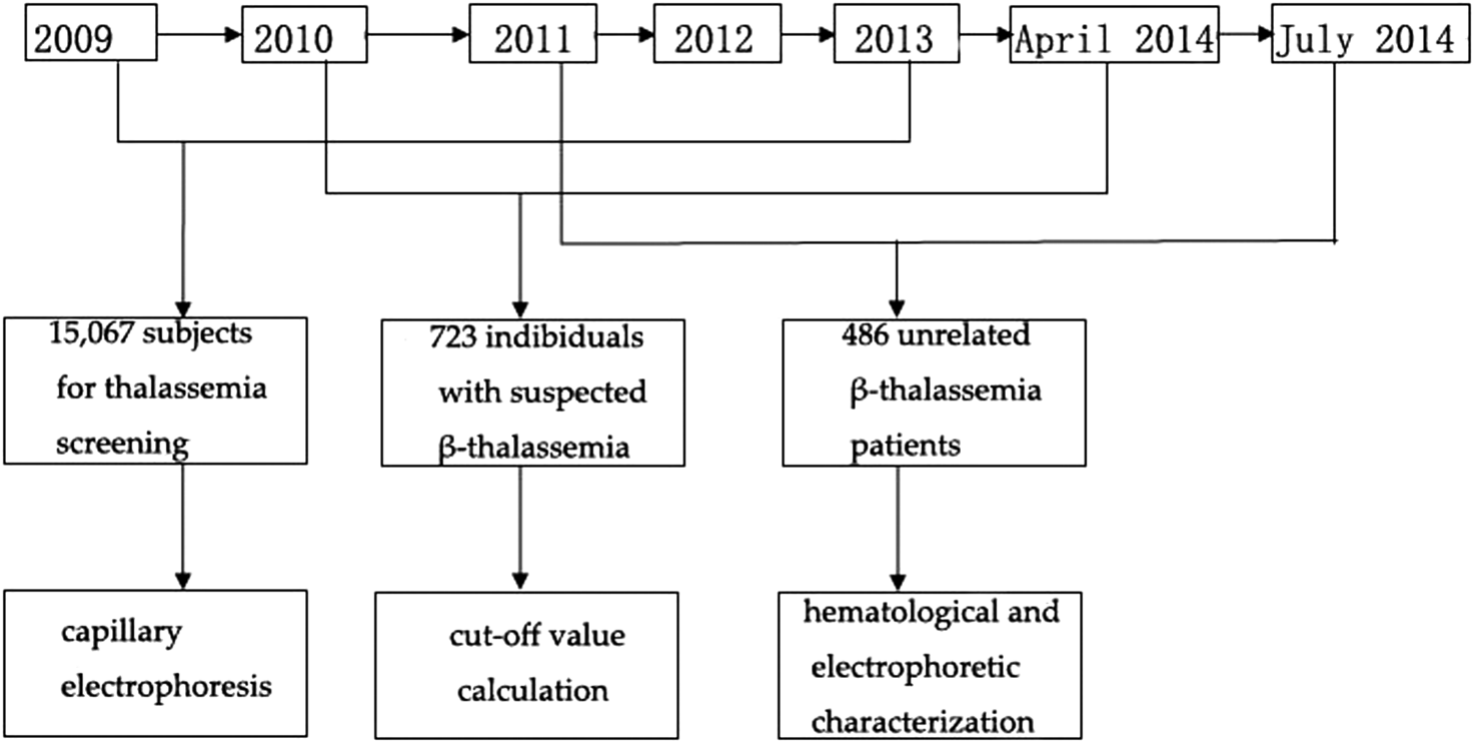
Flow diagram and participant numbers in this study. Capillary electrophoresis, cut-off value calculation, haematological and electrophoretic characterisation of β-thalassaemia mutation were three independent studies conducted during different time periods.

In this study, we performed a retrospective analysis using capillary electrophoresis to determine the HbA_2_ data set, calculated the diagnostic criteria for β-thalassaemia and analysed haematological parameters to provide an additional characterisation of this disease. There were three independent studies conducted during different time periods. The study design is shown in figure 1.

### Setting

This study was conducted in The First Peoples' Hospital of Yunnan Province. This hospital is located in the Jinbi road of Kunming city. It is a general hospital whose annual clinic amount exceeds 1.6 million people with 2000 beds.

### Participants

Participants for three independent studies were collected during three different time periods: (1) a total of 15 067 subjects (3678 men and 11 389 women, 18–45 years of age) who sought thalassaemia screening during September 2011 to July 2014 and had capillary electrophoresis performed; (2) individuals (n=723) with suspected β-thalassaemia (HbA_2_≥3.5%) or abnormal haemoglobin variants were randomly selected from October 2010 to April 2014 (210 men and 513 women, 18–45 years of age) for cut-off value calculation; (3) unrelated heterozygous β-thalassaemia patients (n=486: 151 men and 335 women, 19–58 years of age), were included in the study to analyse haematological and biochemical parameters between June 2009 to April 2013. Written and informed consent were obtained from the patients. The exclusion criteria were: (1) incomplete information, (2) consanguinity, (3) lack of informed consent and (4) children (below the age of 18 years).

### Capillary electrophoresis

Venous blood samples were collected from subjects (n=15 067) in tubes containing EDTA. Haemoglobin analysis was performed using capillary electrophoresis (Sebia, Paris, France). Internal quality control was performed by analysing the control materials provided by the manufacturer. Mean comparisons were made among HbA_2_ and HbA by gender, comparing men versus women in each of three age groups: 18–45, 20–29 and 30–39 years using t-test.

### DNA extraction and detection of β-globin mutations

DNA was extracted from whole blood using the blood DNA extraction system (Tianlong Bioscience Shenzhen, Xian, China). Detection of 17 known β-globin gene mutations (97.3% of known β-thalassaemia alleles in Chinese populations) for 723 individuals with suspected β-thalassaemia or abnormal haemoglobin variants were performed by polymerase chain reaction (PCR)-reverse dot-blot method as previously described.^10^ These 17 β-globin mutations are as follows: CD 41-42 (–TCTT), intervening sequence (IVS)-II-654 (C>T), −28 (A>G), CD 71/72 (+A), CD 17 (A>T), HbE (CD 26, G>A), CD 31 (–C), CD 27/28 (+C), CD 43 (G>T), -32 (C>A), -29 (A>G), 30 (T>C), CD 14/15 (+G), Cap+40 to+43 (–AAAC), Initiation CD (T>G), IVS-I-1 (G>T) and IVS-I-5 (G>T). Samples without detected mutations were sequenced on an ABI 3700 automated sequencer using primers that ﬂanked the entire β-globin gene, as previously described.^10^

### Cut-off value calculation

Sensitivity, specificity, Youden's Index (YI), likelihood ratio positive (LRP) and likelihood ratio negative (NRP) of HbA_2_ measurements within the interval of 3.5–4.5% were evaluated. A receiver operating characteristic (ROC) analysis was performed to calculate the area under the curve (AUC). In addition, we calculated the cut-off value with the greatest accuracy to identify β-thalassaemia in our population.

### Characterisation of β-thalassaemias

Complete blood counts were performed on 486 unrelated β-thalassaemia heterozygous patients that were diagnosed in our centre using an automated cell counter (Sysmex, Tokyo, Japan). Histograms and tables of descriptive statistics of Hb, MCV, MCH, HbA and HbA_2_ were generated and compared by gender (n>15). Also, HbA, HbA_2_, MCV, MCH and Hb were compared among different genotypes of β-thalassaemia (n>10). Continuous variables were compared using analysis of variance. For multiple comparisons, a *post hoc* analysis was used when appropriate. Duncan's multiple range test was used to decrease type I error rates. All reported p-values are two-sided and were statistically significant if p<0.05. All statistics in this study were computed with SPSS V.16 for Windows.

## Results

### Subjects screened by capillary electrophoresis

The data distribution of HbA and HbA_2_ measurements performed in the study is shown in online supplementary figure S1 and table S1. The mean HbA among the subjects was 96.83% (95% CI for mean: 96.81% to 96.84%), while the mean HbA_2_ was 2.91% (95% CI for mean: 2.90% to 2.92%). Out of these subjects, the majority had HbA_2_ levels ranging from 2.4 to 3.5% (95.45%, 14 382/15 067), while 337 cases (2.24%, 337/15 067) had HbA_2_ levels <2.4% and 348 cases (2.31%, 348/15 067) had HbA_2_ levels >3.5%. Six subjects lacking an HbA band and five subjects without an HbA_2_ band were identified. The HbF band was present in 4381 subjects (29.08%, 4381/15 067) with a mean percentage of 1.17% (95% CI for mean: 1.02% to 1.33%). There were no significant differences in HbA_2_ levels between male and female in each three age groups (18–45, 20–29 and 30–39) (see online supplementary figure S1). However, HbA levels were significantly higher in men than in women in all three age groups (18–45, 20–29 and 30–39) (p<0.01) (see online supplementary figure S1).

10.1136/bmjopen-2016-013367.supp1 supplementary figure

10.1136/bmjopen-2016-013367.supp2 supplementary tables

### Cut-off value calculation

Among the total 723 specimens investigated for β-globin gene mutations, 22 different mutations were found in 566 cases (78.28%, 566/723), with HbA_2_ levels ranging from 1.8 to 7.9%. These included a total of 563 β-thalassaemia heterozygotes, one haemoglobin E (HbE) homozygosity and two compound heterozygotes (table 1). β-globin mutations were not detected in the remaining 237 subjects. Among β-thalassaemia heterozygotes, HbA_2_ values ranged from 1.8% to 4.0% in 69 of 566 subjects (69/566, 12.19%), while the majority (497/566, 87.81%) had HbA_2_ values ≥4.0%. The sensitivity, speciﬁcity, YI, LRP and NRP of each selected cut-off point in screening for β-thalassaemia are summarised in table 2. Regarding haematological parameters, HbA_2_ at the new cut-off value of 4.0% yielded high values (0.898, 95% CI 0.874 to 0.919) for AUC and YI (0.75) (see online supplementary figure S2). The new cut-off had the highest accuracy, with a sensitivity of 85.16% and a specificity of 89.81%, and is therefore a suitable discriminator for screening of β-thalassaemia in this population. Using the currently established cut-off (HbA_2_ ≥3.5%) only yielded sensitivity and speciﬁcity values of 96.64% and 6.37%, respectively.

**Table 1:** Number of β-globin mutations found in this study

Mutation	Type	n	Number of alleles	Allele frequency (%)
CD 17 (A>T) (HBB:c.52A>T)	β^0^/β^A^	166	166	29.04
CD 41-42 (–TCTT) (HBB:c.126_129delCTTT)	β^0^/β^A^	145	146	26.26
CD 26 (G>A) (HBB:c.79G>A)	β^+^/β^A^	107	110	19.13
IVS-II-654 (C>T) (HBB:c.316-197C>T)	β^+^/β^A^	90	92	16.00
–28 (A>G) (HBB:c.-78A>C)	β^+^/β^A^	20	20	3.48
CD 71/72 (+A) (HBB:c.216_217insA)	β^0^/β^A^	12	12	2.09
CD 27/28, +C (HBB:c.84_85insC)	β^0^/β^A^	7	7	1.22
IVS-I-1 (G>T) (HBB:c.92+1G>T)	β^+^/β^A^	5	5	0.87
IVS-I-5 (G>C) (HBB:c.92+5G>C)	β^+^/β^A^	1	1	0.17
CD 5 (–CT) (HBB:c.17_18delCT)	β^0^/β^A^	1	1	0.17
Hb Dieppe, CD 127 (A>G) (HBB:c.383A>G)	β^0^/β^A^	1	1	0.17
Initiation CD (T>C) (HBB:c.2T>C)	β^0^/β^A^	1	1	0.17
CD121 (G>T) (HBB:c.364G>T)	β^0^/β^A^	1	1	0.17
–31 (A>C) (HBB:c.-81A>G)	β^+^/β^A^	1	1	0.17
–29 (A>G) (HBB:c.-79A>G)	β^+^/β^A^	1	1	0.17
CD 43 (G>T) (HBB:c.130G>T)	β^0^/β^A^	1	1	0.17
CD 113 (T>A) (HBB:c.341T>A)	HbVar	1	1	0.17
CD 22 (A>C) (HBB:c.68A>C)	HbVar	1	1	0.17
CD 47 (G>A) (HBB:c.142G>A)	HbVar	1	1	0.17
CD 41-42/IVS-II-654	β^0^/β^0^	1	–	–
IVS-II-654/CD 26	β^0^/β^+^	1	–	–
CD 26/CD 26	β^+^/β^+^	1	–	–
Total number of alleles	–	566	569	100

β^0^, production of β-globin chain is entirely eliminated; β^+^, production of β-globin chain is reduced; HBB, beta globin; Hb Dieppe, haemoglobin Dieppe; HbVar, haemoglobin variant.

**Table 2:** Predictive value of evaluated indices of the ROC analysis for β-thalassaemia

HbA_2_ (%)	TP	FN	FP	TN	LRP	NRP	Sn	Sp	YI
3.5	547	147	19	10	1.03	0.53	96.64	6.37	0.03
3.6	540	85	26	72	1.76	0.10	95.41	45.86	0.41
3.7	531	49	35	108	3.01	0.09	93.82	68.79	0.63
3.8	512	35	54	122	4.06	0.12	90.46	77.71	0.68
3.9	497	25	69	132	5.52	0.14	87.81	84.08	0.72
**4.0**	**482**	**16**	**84**	**141**	**8.34**	**0.17**	**85.16**	**89.81**	**0.75**
4.1	473	15	93	142	8.78	0.18	83.57	90.45	0.74
4.2	466	13	100	144	9.94	0.19	82.33	91.72	0.74
4.3	456	13	110	144	9.73	0.21	80.57	91.72	0.73
4.4	449	12	117	145	10.38	0.22	79.33	92.36	0.72
4.5	445	12	121	145	10.29	0.23	78.62	92.36	0.71

Likelihood ratio positive (LRP): sensitivity ÷ (1−specificity); likelihood ratio negative (NRP): (1−sensitivity)/specificity. Youden's Index (YI), sensitivity (Sn): true positive ÷ (true positive+false negative); specificity (Sp): true negative ÷ (true negative+false positive).

FN, false negative; FP, false positive; TN, true negative; TP, true positive.

Bold signifies HbA2 at the cut-off value of 4.0% yielded high values (0.898, 95% CI 0.874 to 0.919) for AUC and YI.

### Haematological and electrophoretic characterisation of β-thalassaemia mutation

**Figure 2:**
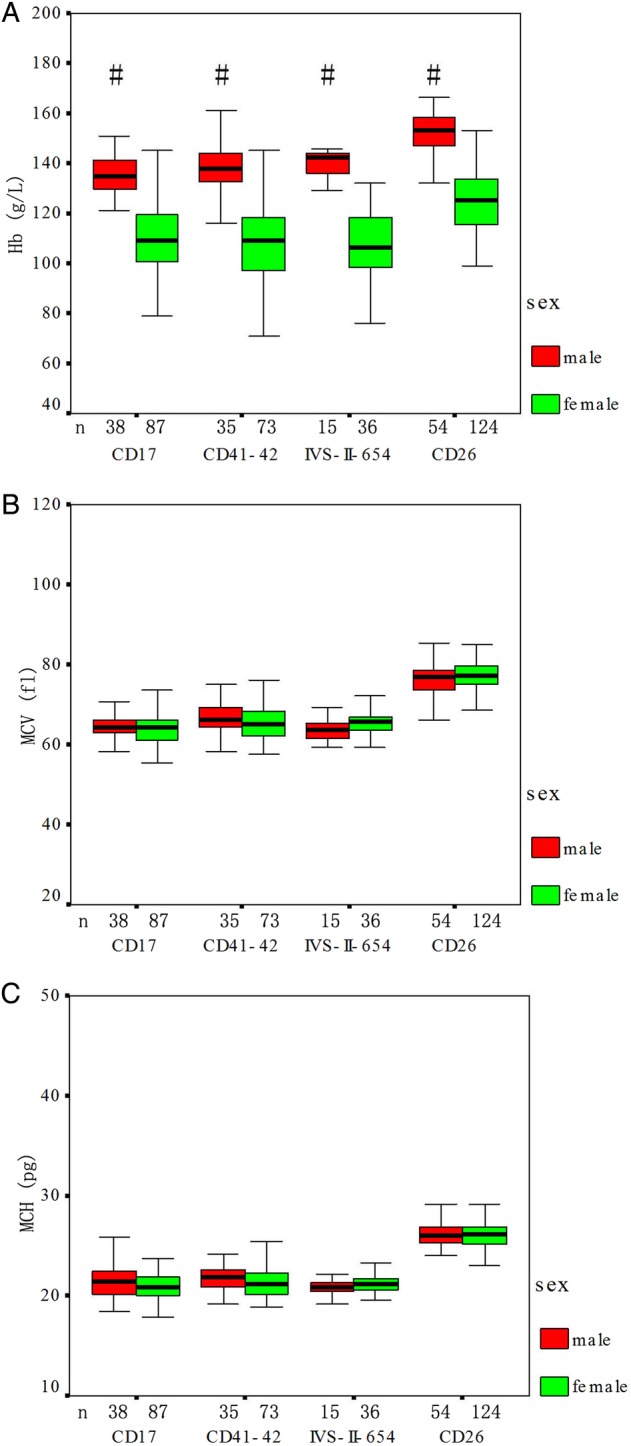
Haematological characterisation of β-thalassaemia according to sex. # Men have significantly higher Hb values (p<0.05) than women. No differences were observed between genders for MCV and MCH. (n=486 total unrelated heterozygous β-thalassaemia cases). Hb, haemoglobin; MCH, mean cell haemoglobin; MCV, mean cell volume.

**Figure 3:**
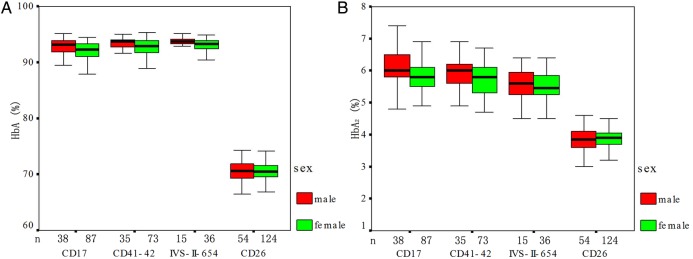
Electrophoretic characterisation of β-thalassaemia according to sex in the current study. No differences were observed between genders for HbA and HbA_2_ (n=486 total unrelated heterozygous β-thalassaemia). HbA, haemoglobin alpha; HbA2, haemoglobin alpha 2.

As shown in tables 3 and online supplementary S2, haematological and molecular characteristics of 486 unrelated heterozygous β-thalassaemia mutations were demonstrated. The mean values for haematological and electrophoretic indices (HbA, HbA_2_, Hb, MCV and MCH) among participants with different genotypes of thalassaemia are shown in table 3. A signiﬁcant difference between genders was observed for Hb, with men having higher Hb compared with the women (p<0.05), whereas no statistically significant differences were found for MCV, MCH, HbA and HbA_2_ values between the two genders (figures 2 and 3). The HbE group showed comparatively higher values of haematological indices (Hb, MCV and MCH) than the other four genotypes of heterozygous β-thalassaemia groups, and −28 (A>G) (HBB (beta globin):c.−78A>C) had signiﬁcantly higher HbA_2_ values compared with other β-thalassaemias (table 3).

**Table 3:** Characterisation of eight types of β-thalassaemia and comparison (mean±SD)

Mutation (*n*)	Hb (g/L)	MCV (fl)	MCH (pg)	HbA (%)	HbA_2_ (%)
CD 17^1^ (125)	118.26±19.14	64.78±6.98	21.52±2.67	92.08±2.24	5.91±0.54
CD 41-42^2^ (108)	117.78±19.54	65.84±5.29	21.68±2.56	92.38±2.93	5.75±0.60
CD 26^3^ (178)	133.04±16.67	76.99±4.52	26.06±1.55	70.67±2.28	3.87±0.44
IVS-II-654^4^ (51)	116.57±19.11	64.81±3.93	21.17±1.06	93.06±1.62	5.55±0.45
−28^5^ (11)	125.64±16.11	73.45±4.97	23.61±1.23	92.49±0.68	6.22±0.47
CD 27-28^6^ (5)	106.00±18.61	68.54±2.26	22.22±0.59	88.56±5.09	5.28±0.56
CD 71-72^7^ (5)	108.80±8.58	64.64±2.60	21.14±0.82	92.76±0.56	5.92±0.29
IVS-I-1^8^ (3)	127.00±13.23	72.40±15.98	25.07±6.51	94.20±0.66	5.73±0.57
Comparisons (p<0.05)	1 vs 3	1 vs 3	1 vs 3	1 vs 3	1 vs 2
	2 vs 3	1 vs 5	1 vs 5	1 vs 4	1 vs 3
	3 vs 4	2 vs 3	2 vs 3	2 vs 3	1 vs 4
		2 vs 5	2 vs 5	3 vs 4	2 vs 3
		3 vs 4	3 vs 4	3 vs 5	2 vs 4
		3 vs 5	3 vs 5		2 vs 5
		4 vs 5	4 vs 5		3 vs 4
					3 vs 5
					4 vs 5

p Value of significant differences (p<0.05) between various five types of β-thalassaemia is listed (n>10). Non-listed comparisons are not significant.

Hb, haemoglobin; HbA, haemoglobin alpha; HbA2, haemoglobin alpha 2; MCV, mean cell volume; MCH, mean cell haemoglobin.

## Discussion

β-Thalassaemia is one of the most common genetic disorders worldwide, and each ethnic group has a mutation set with considerable haematological variations. Clinical features and haematological parameters in β-thalassaemia patients vary with race, lifestyle and altitude. In China, CD 17 (A>T) (HBB:c.52A>T), CD 41-42 (-TCTT) (HBB:c.126_129delCTTT), -28 (A>G) (HBB:c.-78A>C), IVS-II-654 (C>T) (HBB:c.316-197C>T), HbE (CD 26, G>A) (HBB:c.79G>A) and CD 71/72 (+A) (HBB:c.216_217insA) mutations account for more than 90% of all β-thalassaemia mutations in the Chinese population.^2^ Characterisation and screening of these mutations provides a database to aid in the prevention and control of thalassaemia. Samples for participants used for capillary electrophoresis, cut-off value calculation and characterisation of β-thalassaemia were not collected at the same time and this is a limitation of this study. There are very few studies describing the detection and quantification of electrophoretic and haematological parameters for screening thalassaemia in the Chinese population. Thus, our report is the first characterisation of β-thalassaemia mutations in people of Southwestern China and will serve as a key reference for this population.

The majority of individuals screened in this study were women, since patients seeking prenatal diagnosis and genetic counselling represent a large portion of the study participants. Six subjects lacking an HbA band and five subjects without an HbA_2_ band were identified. Among these subjects, four were HbE homozygous, one was β-thalassaemia/HbE compound heterozygous and one had a haemoglobin variant mutation. HbA levels were significantly higher in male than female subjects in the three age groups (see online supplementary figure S1). However, no signiﬁcant difference was observed in HbA_2_ in all age groups. In our study, most of the subjects (95.45%, 14 382/15 067) had HbA_2_ levels that were within a narrow range (2.4–3.5%), similar to previous observations in Nigerian patients.^11^

Carriers of β-thalassaemia have increased HbA_2_ values. Of the 723 subjects analysed, the β-globin gene defect was identified in 78.28% of cases (566/723; range 1.8–7.9%). One case without the β-globin mutation had the highest HbA_2_ value (7.9%), which may be due to a haemoglobin variant coeluting with HbA_2_ peak.^12^ Only one case of β-thalassaemia major was found in this study, which was due to the fact that children were excluded. The majority of β-thalassaemia major patients die before reaching the age of 5.^13^

Having an HbA_2_ level >3.5% is the current diagnostic criterion for the β-thalassaemia trait,^14^ while values >4.0% are also used to identify β-thalassaemia carriers in some regions.^15^ In this study, a higher cut-off point of HbA_2_ levels (HbA_2_ ≥4.0%) determined by ROC curves had the highest accuracy in identifying β-thalassaemia carriers. This result differed from previous reports,^16–18^ which may be explained by regional differences in the spectrum and cut-off values for β-thalassaemia mutations. In our study, most β-thalassaemia mutations (497/566, 87.81%) had a mean HbA_2_ exceeding 4.0%, while silent β-thalassaemia mutations with near normal haematological indices and borderline HbA_2_ values (3.5–4.0%) were rare in our population. Therefore, the diagnostic thresholds should be increased for this population.

The cut-off point was established for all samples, including those from β-thalassaemia patients and those having abnormal haemoglobins. And Hb variant fraction could be easily screened by abnormal band for capillary electrophoresis.^19^ In Yunnan, HbE (CD 26, G>A) (HBB:c.79G>A), an abnormal haemoglobin, is the most prevalent β-globin gene mutation (with a frequency of 30.5%),^20^ with comparatively lower HbA_2_ values. Here, the mean percentage of HbA_2_ in patients with HbE was 3.87±0.44, which was similar to that reported for Americans.^21^ HbE and other abnormal haemoglobins, such as CD 22 (A>C) (HBB:c.68A>C) (HbG-Coushatta), have been reported to decrease the expression of HbA_2_.^22^ In this study, most β-thalassaemia were characterised by increased HbA_2_ values ranging from 4.3% to 6.6% (93.86%, 428/456).

Subjects with a β-globin mutation usually have lower Hb and erythrocyte indices compared with those without an identifiable mutation^23^ and the clinical and haematological parameters in patients having β-thalassaemia are widely variable.^24^ This study was conducted in Kunming, the capital of Yunnan province, which has an altitude of 1895 m. Regional altitude differences are a contributing factor in the variation of these parameters, as high altitudes are known to elevate Hb values. Men had higher Hb values in all five genotype groups compared with women, whereas, no statistical sex-based significant differences were observed for MCV, MCH, HbA and HbA_2_ values. These results were similar to previously reported data.^25^ HbE (CD 26, G>A) (HBB:c.79G>A) is clinically asymptomatic with minimal erythrocyte morphological abnormalities in heterozygous individuals; this type had the highest MCV, MCH and Hb values in this study. Subjects with the −28 (A>G) (HBB:c.−78A>C) mutation (β^+^-thalassaemia), a mutation in the TATA box of the proximal promoter region, had higher MCH, MCV and HbA_2_ values compared with other β-thalassaemia types (CD 17 (A>T) (HBB:c.52A>T), CD 41-42 (-TCTT) (HBB:c.126_129delCTTT) and IVS-II-654 (C>T) (HBB:c.316-197C>T)). The −28 (A>G) (HBB:c.−78A>C) phenotype is similar to other β-globin gene promoter mutations.^26^

In conclusion, we offered a retrospective analysis of β-thalassaemia that can be used for prenatal diagnosis, genetic counselling, thalassaemia control and screening. Characterisation of β-thalassaemia and diagnostic thresholds for β-thalassaemia can be used to identify patients more precisely.
