# Supplementary material for: Haematological and electrophoretic characterisation of β-thalassaemia in Yunnan province of Southwestern China
Source: BMJ Open. 2017 Jan 31;7(1):e013367. doi: 10.1136/bmjopen-2016-013367 (PMC5293871; doi:10.1136/bmjopen-2016-013367)

Figure S1 Distribution of HbA2 (A) and HbA (B) in all samples. The data distribution of HbA and HbA2 were measured according to age. # Males show significantly higher Hb A values ($p < 0.05$) than females in three age group (18–45, 20–29, and 30–39 years). There was no significant differences in HbA2 levels in all three age groups. The sample size of the other two age categories (<20, 40-45) were small, which was not suitable to compare.

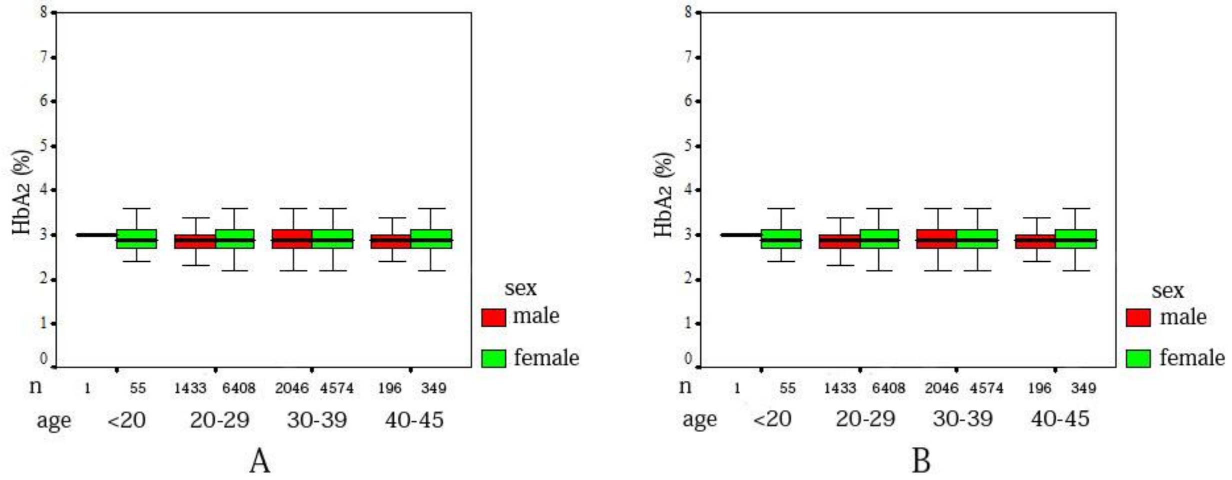


Figure S2 Receiver operative characteristic curves (ROC) of the cut-off point calculation. A: cut-off value at HbA2 4.0%. HbA2 at the new cut-off value of 4.0% yielded high values (0.898, 95% CI: 0.874 to 0.919) for AUC and YI (0.75).

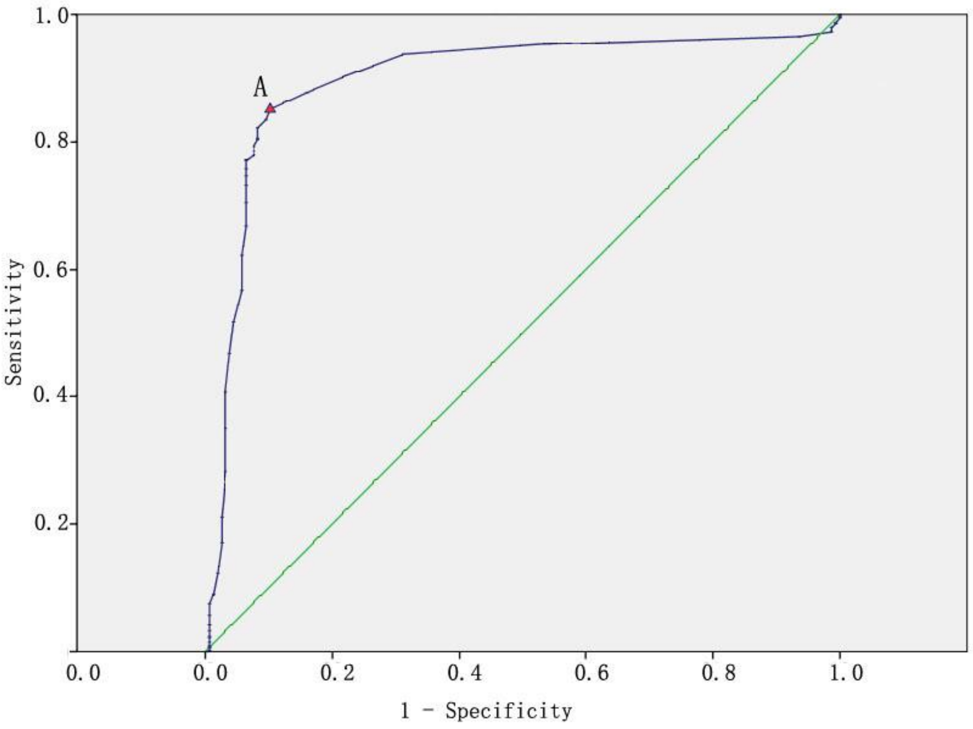

Supplement: supplementary figure [file bmjopen-2016-013367supp_figures.pdf]
