# Supplementary material for: Haematological and electrophoretic characterisation of β-thalassaemia in Yunnan province of Southwestern China
Source: BMJ Open. 2017 Jan 31;7(1):e013367. doi: 10.1136/bmjopen-2016-013367 (PMC5293871; doi:10.1136/bmjopen-2016-013367)
Supplement: supplementary tables [file bmjopen-2016-013367supp_tables.pdf]

Table S1 Distribution of HbA and HbA_2 in all samples

Parameters	Sex	Age	n	Mean (%)	SD (%)	95% CI for mean
HbA	Male	18–45	3675	96.95	0.74	96.93%–96.97%
		<20	1	-	-	-
		20–29	1433	96.98	0.59	96.95%–97.01%
		30–39	2045	96.92	0.85	96.88%–96.96%
		40-45	196	97.02	0.50	96.95%–97.09%
	Female	18–45	11386	96.79	1.01	96.77%–96.81%
		<20	55	96.92	0.55	96.77%-97.07%
		20–29	6408	96.79	1.02	96.77%–96.82%
		30–39	4574	96.78	1.01	96.75%–96.81%
		40-45	349	96.71	0.95	96.61%–96.84%
	total	18–45	15061	96.83	0.96	96.81%–96.84%
HbA_2	Male	18–45	3676	2.91	0.42	2.90%–2.92%
		<20	1	-	-	-
		20–29	1433	2.90	0.34	2.88%–2.92%
		30–39	2046	2.92	0.44	2.90%–2.94%
		40-45	196	2.89	0.38	2.84%–2.95%
	Female	18–45	11386	2.91	0.43	2.90%–2.92%
		<20	55	2.91	0.42	2.89%-2.91%
		20–29	6408	2.90	0.42	2.89%–2.91%
		30–39	4574	2.92	0.45	2.90%–2.93%
		40-45	349	2.95	0.47	2.90%–2.96%
	total	18–45	15062	2.91	0.43	2.90%–2.92%

SD: Standard Deviation; CI: confidence interval. HbF was only detected in 29.08%

(4,381/15,067) of the subjects, which was not suitable for the description of mean± SD.

Table S2 Characterization of 7 types of β-thalassemia according to sex (mean ± SD)

Mutation	Sex (n)	Hb (g/L)	MCV (fl)	MCH (pg)	HbA (%)	HbA_2 (%)
CD 17	F (87)	110.30±16.12	64.86±7.96	21.54±3.01	91.80±2.43	5.83±0.52
CD 17	M (38)	136.47±11.55	64.62±4.00	21.46±1.66	92.74±1.55	6.11±0.54
CD 41-42	F (73)	108.71±15.50	65.39±5.76	21.23±1.96	92.04±3.04	5.66±0.57
CD 41-42	M (35)	137.57±11.71	66.82±5.85	22.56±3.74	92.87±2.68	5.87±0.65
CD 26	F (124)	125.18±11.90	77.09±4.74	26.01±1.63	70.64±2.24	3.88±0.45
CD 26	M (54)	151.11±11.07	76.76±4.01	26.18±1.33	70.74±2.37	3.86±0.43
IVS-II-654	F (15)	107.47±14.23	65.35±4.21	21.26±1.13	92.85±1.74	5.53±0.42
IVS-II-654	M (36)	138.40±8.65	63.52±2.90	20.97±0.88	93.57±1.19	5.59±0.53
-28	F (10)	123.5±15.25	72.99±5.00	23.41±1.09	92.49±0.72	6.15±0.44
-28	M (1)*	-	-	-	-	-
CD 27-28	F (5)	106.00±18.61	68.54±2.26	22.22±0.59	88.56±5.09	5.28±0.56
CD 27-28	M (0)*	-	-	-	-	-
CD 71-72	F (4)	105.50±5.07	63.15±2.69	21.45±0.50	92.60±0.50	5.80±0.14
CD 71-72	M (1)*	-	-	-	-	-

*: mean and standard deviation (SD) cannot be calculated (n ＜3).
